# Epithelial–mesenchymal transition related genes in unruptured aneurysms identified through weighted gene coexpression network analysis

**DOI:** 10.1038/s41598-021-04390-6

**Published:** 2022-01-07

**Authors:** Yong’an Jiang, JingXing Leng, Qianxia Lin, Fang Zhou

**Affiliations:** 1 Department of Neurosurgery, Jiangxi Provincial People’s Hospital, Nanchang, 330006 Jiangxi China; 2 Jiangxi University of Traditional Chinese Medicine, Nanchang, 330006 Jiangxi China; 3 Department of Vascular Breast Surgery, Jiangxi Provincial People’s Hospital, Nanchang, 330006 Jiangxi China

**Keywords:** Computational biology and bioinformatics, Molecular biology, Biomarkers, Oncology

## Abstract

Intracranial aneurysm (IA) can cause fatal subarachnoid hemorrhage (SAH) after rupture, and identifying patients with unruptured IAs is essential for reducing SAH fatalities. The epithelial–mesenchymal transition (EMT) may be vital to IA progression. Here, identified key EMT-related genes in aneurysms and their pathogenic mechanisms via bioinformatic analysis. The GSE13353, GSE75436, and GSE54083 datasets from Gene Expression Omnibus were analyzed with limma to identify differentially expressed genes (DEGs) among unruptured aneurysms, ruptured aneurysms, and healthy samples. The results revealed that three EMT-related DEGs (*ADIPOQ*, *WNT11*, and *CCL21*) were shared among all groups. Coexpression modules and hub genes were identified via weighted gene co-expression network analysis, revealing two significant modules (red and green) and 14 EMT-related genes. Gene Ontology and Kyoto Encyclopedia of Genes and Genomes pathway analyses suggested that cytokine interactions were closely related. Gene set enrichment analysis revealed that unruptured aneurysms were enriched for the terms “inflammatory response” and “vascular endothelial growth”. Protein–protein interaction analysis identified seven key genes, which were evaluated with the GSE54083 dataset to determine their sensitivity and specificity. In the external validation set, we verified the differential expression of seven genes in unruptured aneurysms and normal samples. Together, these findings indicate that *FN1*, and *SPARC* may help distinguish normal patients from patients with asymptomatic IAs.

## Introduction

Intracranial aneurysms (IAs), also known as cerebral aneurysms, are characterized by localized swelling of the blood vessel wall, which begins to bulge and become sac-like. Once the aneurysm wall ruptures, it can lead to life-threatening subarachnoid hemorrhage, which usually has a very poor prognosis^1^. These complications affect quality of life, the domestic economy, and could also be life threatening. Usually, unruptured intracranial aneurysms (UIAs) have no clinical symptoms and occur in 2–3% of the population. Currently, there is a lack of effective treatment methods for UIA, and most treatment options are invasive and have a poor prognosis^2^. Therefore, improved screening and clinical evaluation of patients with UIAs are of paramount importance. The popularization of gene therapy and detection methods promise to provide a powerful boost to understanding the mechanisms underlying UIAs.

Recently, various studies have focused on exploring intracranial vascular diseases at the gene level. Macrophages secrete prostaglandin E2 (PE2), thereby activating nuclear factor kappaB (NF-κB) and increasing the expression of macrophage chemotactic protein 1 and COX2, which leads to reshaping of the aneurysm wall^3^. Upon administering anti- stromal cell-derived factor-1 (SDF-1) blocking antibodies to mice and analyzing the components of the aneurysm wall, it was found that SDF1 plays a key role in the angiogenesis of the aneurysm wall and the migration and proliferation of inflammatory cells^4^. Studies have also confirmed that the mechanism of unruptured aneurysms confirmed with immunohistochemical methods may be caused by an imbalance of VEGF (endothelial growth factor receptor), VEGFR1, and VEGFR2 expression and the interaction of VEGF with NO (nitric oxide). However, to date, there have been very few studies on the pathogenesis of UIA, and further research is needed to clarify this.

Cell differentiation is vital at all time points of heart, brain, lung, and kidney vascular development and remodeling. As an important pathological process of disease progression, it is an emergency response to vascular injury. EMT is characterized by the loss of epithelial characteristics, mesenchymal phenotype of epithelial cells, cell contact, and basement membrane attachment. EMT is a key process in wound healing and tissue regeneration. A study by Luo et al.^5^ showed that SO_2_ (sulfur dioxide) is involved in pulmonary artery interstitial thickening and middle layer hypertrophy, and that it inhibits the EMT process. Another study by Li et al.^6^ confirmed that elastase-induced cystic aneurysms are related to stromal cell-derived factor-1α (SDF-1α), and VE-concept proteins may be involved in aneurysm remodeling. Additionally, in rat aneurysm cells, (Matrix Metallopeptidase 2) MMP2 and (Matrix Metallopeptidase 9) MMP9 are involved in aortic aneurysm proliferation and migration^7^. Nakaoka et al.^8^ identified therapeutic targets for unruptured IAs. Landry et al.^9^ demonstrated for the first time the immune mechanism of IA rupture and new drug candidates. Whether EMT plays an important role in the progression of aneurysm is worthy of further exploration.

However, there are still very few studies on the mechanism of UIA that can help identify and treat patients with asymptomatic IA. UIA diagnosis is extremely important for reducing the incidence of subarachnoid hemorrhage (SAH). In this study, we aimed to identify key EMT genes using different bioinformatic methods and explore the underlying mechanisms of UIA.

## Materials and methods

### Epithelial–mesenchymal transition-related genes and microarray data processing

A total of 1184 human EMT-related genes were downloaded from the dbEMT2 database (http://dbemt.bioinfo-minzhao.org/)^10^. The GSE13353^11^, GSE75436^12^, and GSE54083^8^ datasets (information on samples and authors listed in Supplementary Table S1) were downloaded from the GEO database (https://www.ncbi.nlm.nih.gov/gds/), and the probe ID of the corresponding probe matrix was used to annotate the gene symbols to form a gene expression matrix. The GSE54083 dataset was used as the external validation dataset, and the research procedure was carried out step by step according to the flowchart shown in Supplementary Fig. S1.

### Differential expression analysis of epithelial–mesenchymal transition-related genes

Matrix log_2_ transformation was used to standardize data, and the sva package processed the batch effect of the combined sample datasets (GSE13353 and GSE75436). The obtained EMT gene data were merged. The microarray data linear model (limma) package for R was used to identify DEGs; the ruptured (n = 11), unruptured (n = 2 3), and healthy groups (n = 15) were respectively paired to compare the results to acquire DEGs, where *p* < 0.01, |logFC| > cutoff. The DEG cutoff value was as follows: mean (abs(logFC)) + 2*sd (abs(logFC)).

### Construction of weighted gene co-expression network

The WGCNA package^13^ was used to construct gene co-expression networks in the R environment. The merged matrix file and clinical feature file were converted into a format that conforms to WGCNA. Outlier samples were identified through sample clustering, and outlier samples (height = 40), namely GSM1955157, GSM337112, GSM1955165, and GSM1955170, were removed. The soft threshold (β) was calculated from the results of the scale-free topological fit index and the average connectivity. We determined the optimal β value for an R^2^ value > 0.9, and the corresponding suitable average connectivity. A topological overlap measure (TOM) matrix was generated based on the adjacency matrix, and average linkage hierarchical clustering and dynamic tree cutting were performed to detect gene modules. The minimum number of genes was 30 in the modules, and the threshold for merging similar modules was 0.25. We used Pearson correlation analysis to determine the relationship between color modules, trait modules, and key genes.

### Verification and acquisition of hub genes

The central genes in the expression modules were based on eigengene-based module connectivity or module membership (kME) index. Therefore, the calculation of gene expression level and eigengene of the module in the calculation module depended on the size of kME in a specific module; genes with |kME|≥ 0.8 were considered hub genes, and the threshold of gene significance was 0.2.

### Epithelial–mesenchymal transition-related gene functional enrichment analysis

We performed KEGG pathway and GO analyses of the DEGs of interest. We used the “org.Hs.eg.db” package to convert the gene symbols into ENTREZ IDs and the “clusterProfiler” package for GO and KEGG analysis^14^. GSEA of the modular hub genes was performed using the GSEA package. The threshold was set at *p* < 0.05.

### Construction of protein–protein interaction network

The above-mentioned hub genes were input into the STRING database to search for interacting genes, to retrieve and construct protein–protein correlations and exclude hub genes that were not related to PPI, as assessed via Cytoscape_v3.8.2^15^.

### Statistical analysis

R version 4.0.3(R Core Team (2020). R: A language and environmentfor statistical computing. R Foundation for Statistical Computing, Vienna, Austria. URL https://www.R-project.org/) was used to perform data preprocessing, DEG screening, WCGNA, and functional enrichment analysis. Graphpad Prism 8.0.0(One-way ANOVA followed by Dunnett’s multiple comparisons test was performed using GraphPad Prism version 8.0.0 for Windows, GraphPad Software, San Diego, California USA, www.graphpad.com) was used to analyze the expression of the selected genes in different groups. The “pROC” R package calculated the receiver operating characteristic (ROC) curve and the area under the ROC curve (AUC) to evaluate the diagnostic value of the EMT-hub genes.

## Results

### Identification of epithelial–mesenchymal transition-related differentially expressed genes in samples

**Figure 1:**
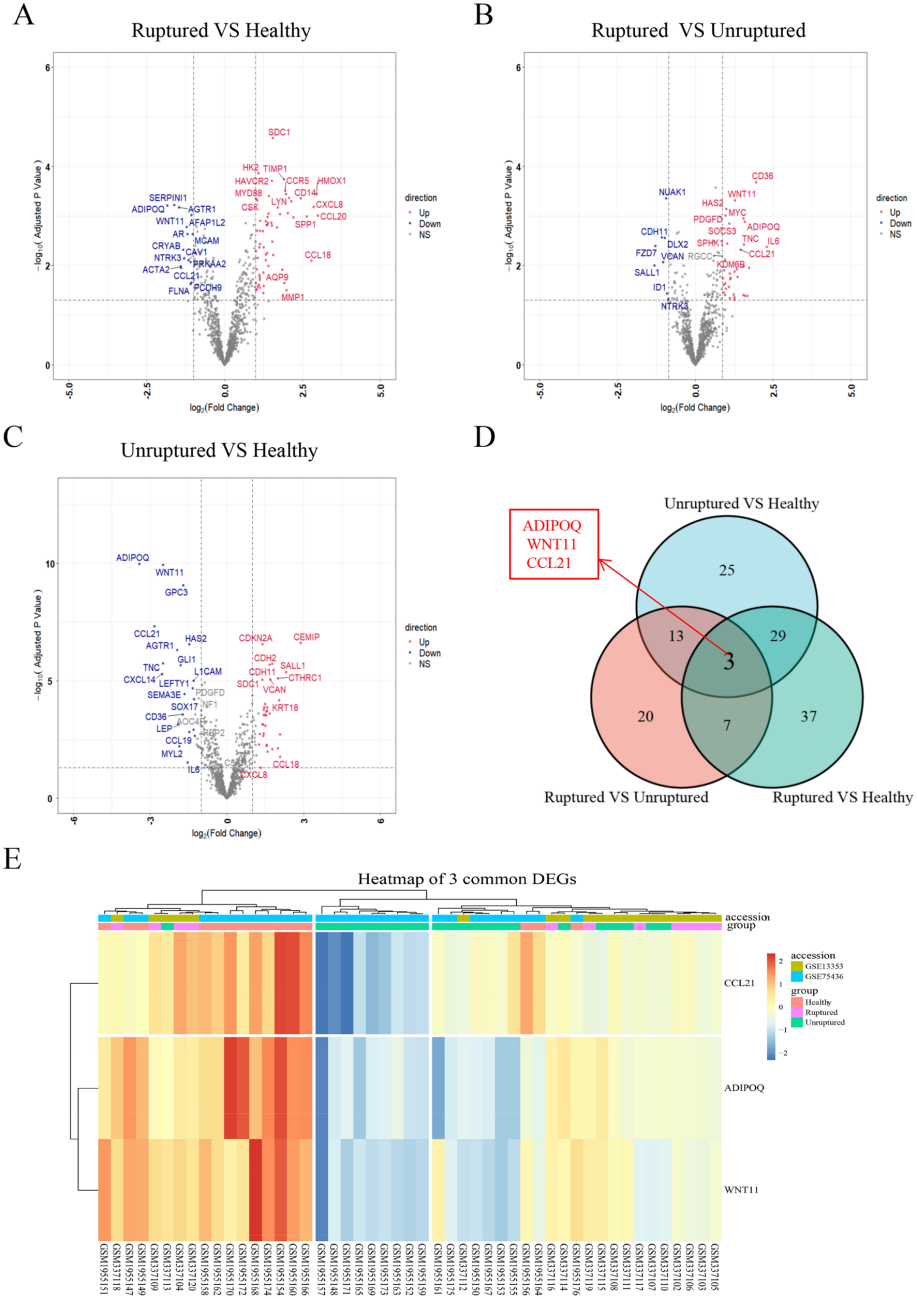
Sample differential gene and expression analysis. (**A**) Epithelial–mesenchymal transition (EMT)-related differentially expressed genes (DEGs) of the ruptured and healthy groups. (**B**) EMT-DEGs of the ruptured and unruptured groups. (**C**) EMT-DEGs of the unruptured and healthy groups. (**D**) Common genes among the three groups (ruptured, unruptured, healthy). (**E**) Relative expression levels of three genes (*ADIPOQ*, *WNT11*, and *CCL21*) in the merged dataset samples. DEGs (differentially expressed genes), up (red): upregulated genes, down (blue): downregulated genes. NS (black): no significance. Above all figures were visualized by R software 4.0.3.

After standardizing the GSE13353 and GSE75436 datasets and de-batch effect (Fig. S2A–D), we analyzed the expression of EMT-related genes in 11 ruptured aneurysm samples (ruptured), 23 unruptured aneurysm samples (unruptured), and 15 healthy patient samples (healthy). Three groups of samples were compared in pairs to identify EMT-related up- and downregulated genes (Fig. 1A–C; Table S2) (ruptured vs. healthy, upregulated: n = 61, downregulated: n = 15; ruptured vs. unruptured, upregulated: n = 35, downregulated: n = 8; unruptured VS. healthy, upregulated: n = 40, downregulated: n = 30). We obtained three common genes (*ADIPOQ, WNT11,* and *CCL21*); the expression patterns of these three genes in the above-mentioned datasets and samples are shown in a heatmap in Fig. 1D, E.

### Gene Ontology enrichment analysis and Kyoto Encyclopedia of Genes and Genomes pathway summary

**Figure 2:**
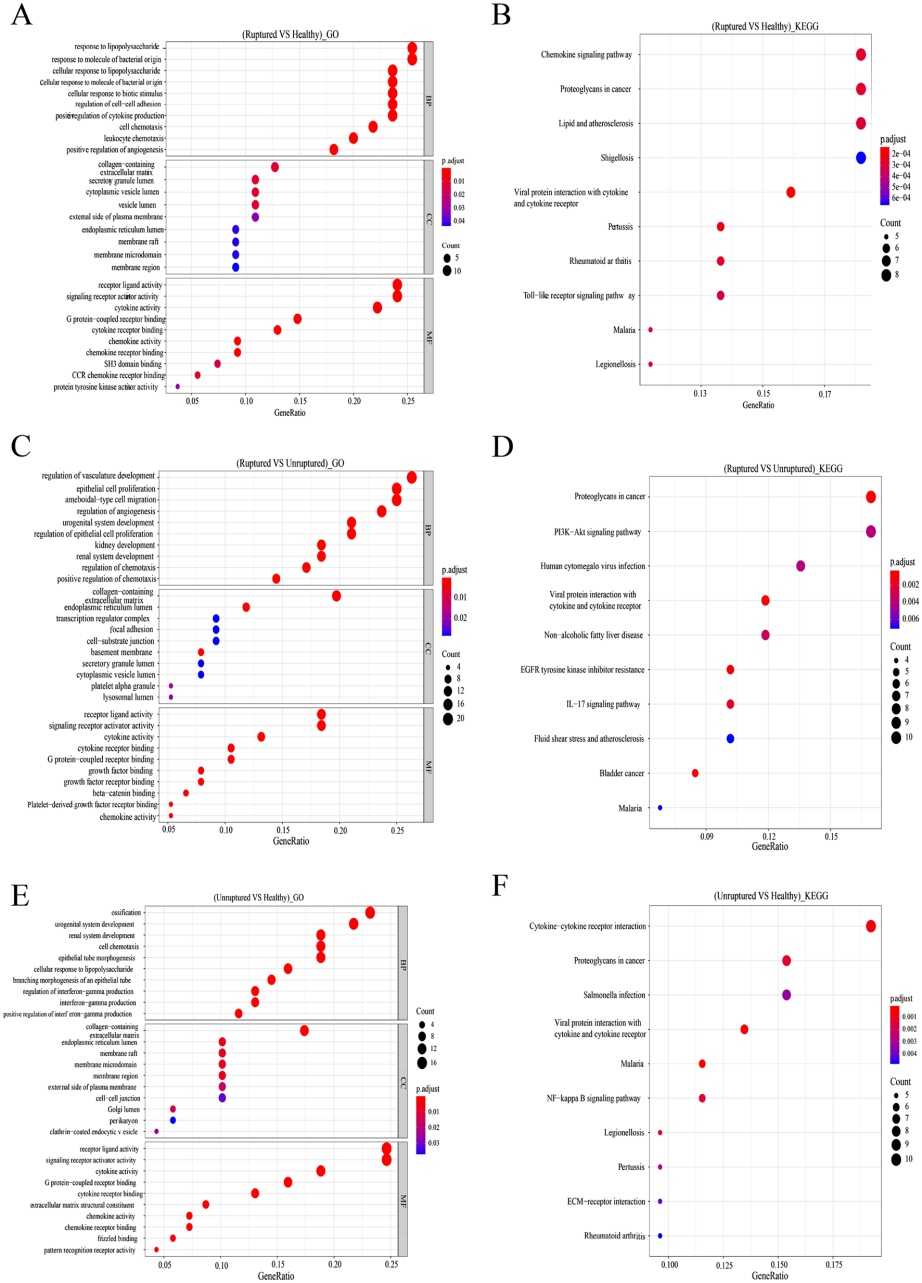
Enrichment and pathway analyses of various DEGs. (**A, B**) Gene Ontology (GO) and Kyoto Encyclopedia of Genes and Genomes (KEGG) analyses of DEGs (ruptured vs. healthy). (**B, C**) GO and KEGG analyses of DEGs (Ruptured vs. Unruptured). (**D, E**) GO and KEGG analyses of DEGs (unruptured vs. healthy). BP (biological process), CC (cellular component), MF (molecular function).above all figures were visualized by R software 4.0.3.

We used Gene Ontology (GO) and Kyoto Encyclopedia of Genes and Genomes (KEGG) analyses to explore the functions and participants of DEGs in ruptured, unruptured, and healthy groups, as well as to understand their mechanisms. The results suggested that the terms “response to lipopolysaccharide” and “response to bacterial molecules” were enriched in ruptured vs. healthy groups (Fig. 2A, B) and participated in the chemokine signaling pathway and proteoglycans in cancer. The terms “Regulation of vasculature development and epithelial cell proliferation,” “proteoglycans in cancers,” and “the PI3K-AKT signaling pathway” were enriched in the ruptured and unruptured groups (Fig. 2C, D). Similarly, in the unruptured vs. healthy groups (Fig. 2E, F), the terms “ossification,” “urogenital system development,” and “the cytokine-cytokine receptor interaction pathway” were enriched.

### Establishment of the weighted gene co-expression network of epithelial–mesenchymal transition-related genes

**Figure 3:**
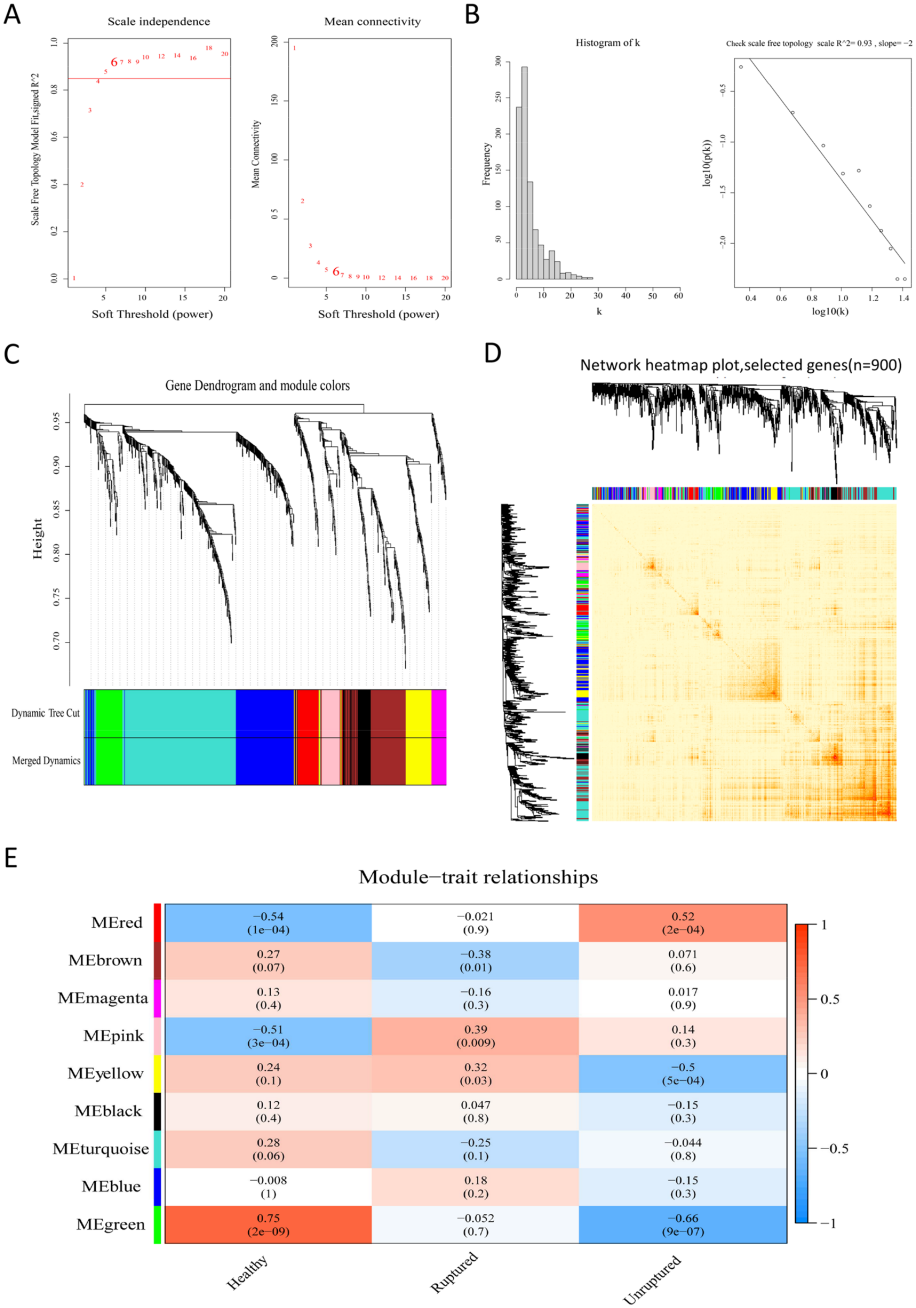
Co-expression network analysis of the top 900 EMT-related genes. (**A**) The scale-free fit index and average connectivity verification of weighted gene co-expression network analysis (softpower = 6). (**B**) When softpower = 6, the connectivity and linear relationship were visualized (R^2^ = 0.93, slope = − 2). (**C**) Based on the difference cluster analysis of consensus topology overlap, the gene dendrogram was obtained with the corresponding module color indicated by the color row. The color modules contained highly connected genes. We set a threshold of 0.25 for the dynamic cut tree merging module. Nine color modules were identified. (**D**) Hierarchical clustering was used to visualize the gene network (TOM plot). (**E**) According to Pearson’s correlation analysis, the correlations between color modules and traits were revealed (unruptured, ruptured, and healthy groups). Red represents positive correlation and blue represents negative correlation. Above all figures were visualized by R software 4.0.3.

**Figure 4:**
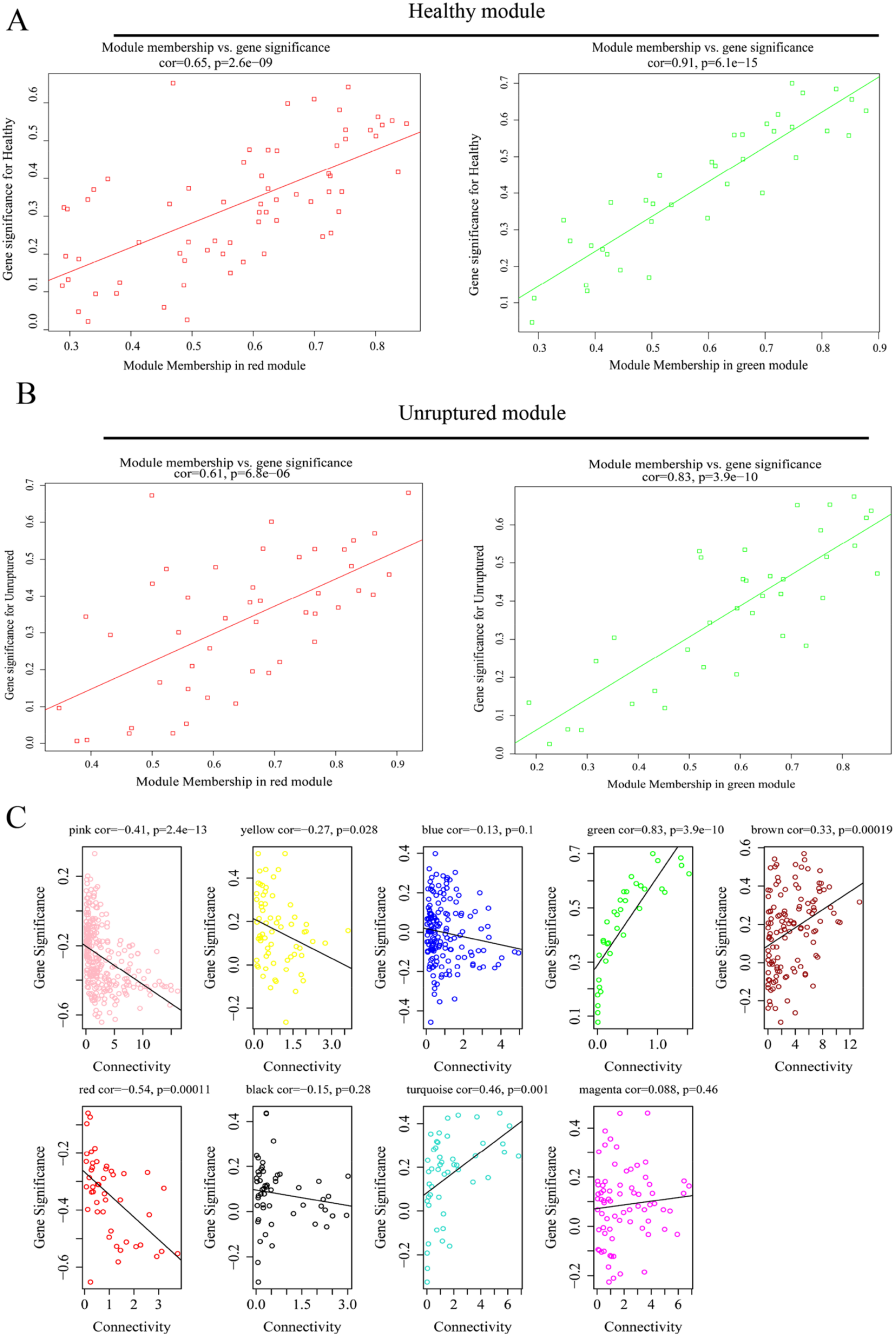
Correlation between traits (unruptured, healthy) and genes in the module and the gene internal connection degree. (**A, B**) Pearson’s correlation analysis of the correlation between traits (unruptured and healthy groups) and genes in the module. (**C**) The degree of gene connectivity in the top 9 color modules. Above all figures were visualized by R software 4.0.3.

Sample clustering screened out outliers (Fig. S3A). EMT-related genes (n = 1184) were ranked according to variance, and the top 900 were selected for (weighted gene co-expression network analysis) WGCNA. After calculating the scale-free model topology fit (R-square) and mean connectivity, β = 6 was selected as the soft threshold (R^2^ = 0.93) (Fig. 3A, B) to ensure a scale-free network. The module feature vector correlation coefficient > 0.75 was merged (Fig. 3C and Fig. S4A) based on the dissimilarity measure (1-TOM), and the dendrogram of the above-mentioned EMT-related genes was clustered (Fig. 3D). Through hierarchical clustering to identify nine important color modules, Spearman correlation analysis was used to determine the relationship between color modules and traits and to identify which modules were independent of each other (Fig. 3E and Fig. S4B, C). Interestingly, the green (r = 0.75, *p* = 2e−09) and red (r =  − 0.54, *p* = 1e−04) modules were positively and negatively correlated with the healthy group, respectively. Furthermore, the green (r =  − 0.66, 9e−07) and red (r = 0.52, *p* = 2e−04) modules were negatively and positively correlated to the unruptured group, respectively. We believed that the red and green modules were the most critical modules of the healthy and unruptured groups. Similarly, the results within modules also showed that the genes in the green and red modules were significantly related to healthy and unruptured groups (Fig. 4A, B). Next, we assessed the relationship between the degree of internal connection of genes and the color modules (Fig. 4C) and found that the internal connection degree of the red and green module genes was related to the red and green color modules (green cor = 0.83, *p* = 3.9e−10; red cor =  − 0.54, *p* = 0.00011).

### Key module enrichment analysis and protein–protein interaction network construction

**Figure 5:**
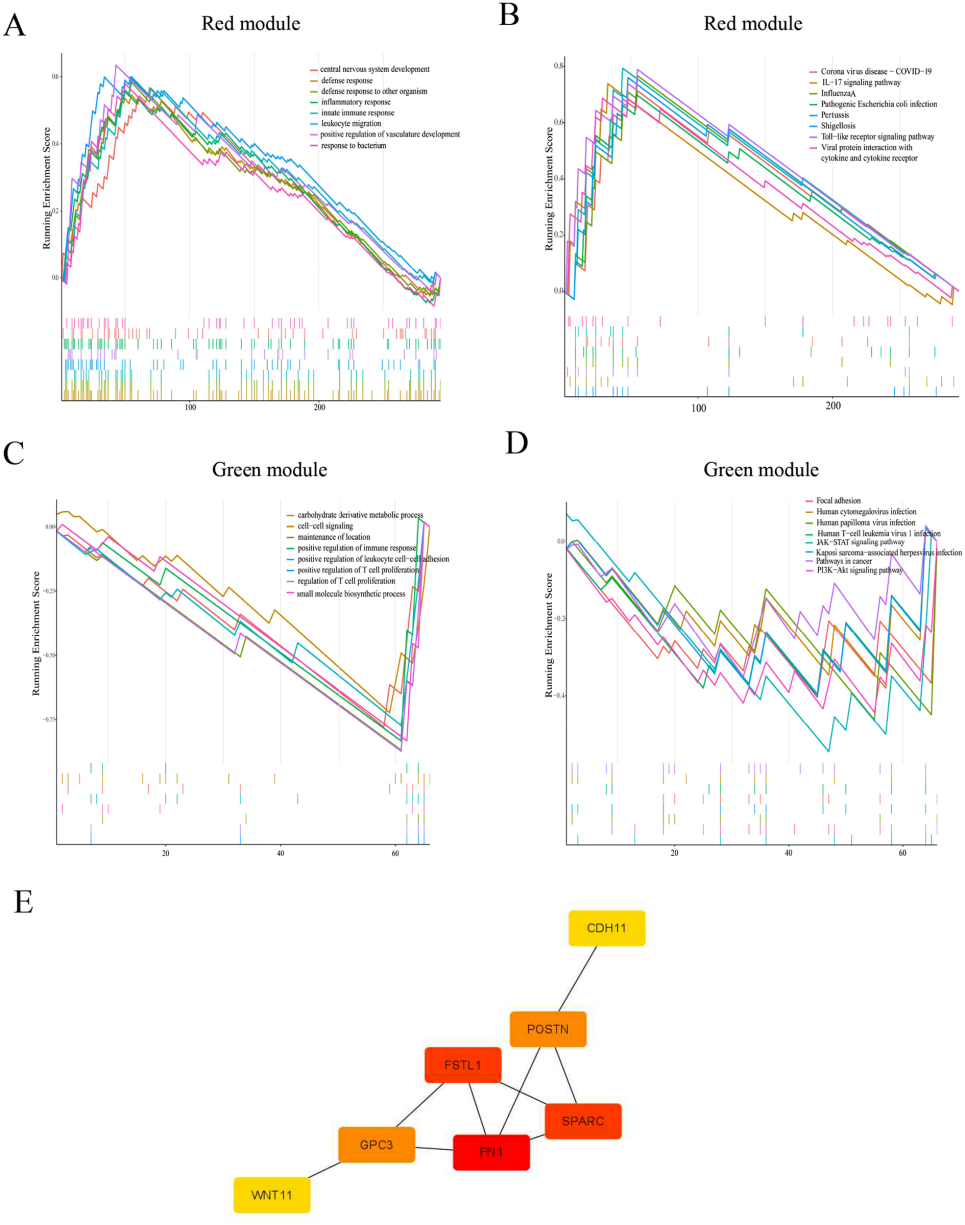
Gene set expression analysis (GSEA) of red and green modules and protein–protein interaction (PPI) network construction. (**A**) GSEA results of the red module biological processes (BP). (**B**) GSEA analysis of red module (Kyoto Encyclopedia of Genes and Genomes [KEGG]). (**C**) GSEA analysis of the green module (BP). (**D**) GSEA result of the green module (KEGG). (**E**) PPI network construction of EMT hub genes via cytoscape; the darker the color, the higher the connectivity. **A**–**D** were visualized by R software 4.0.3 and **E** were visualized via Cytoscape_v3.8.2.

Gene set enrichment analysis (GSEA) was used to explore the potential biological functions of genes in the red and green modules and the pathway mechanisms involved. Results of GSEA analysis showed that the red module biological pathways (BPs) included responses to bacteria and positive regulation of vasculature development (Fig. 5A–D). The results of important pathways involved in KEGG included viral protein interactions with cytokine and cytokine receptors and the Toll-like receptor signaling pathway. The biological processes (BPs) of the green module were primarily the small molecular biosynthetic process and regulation of T cell proliferation. The results of KEGG analysis revealed involvement of the PI3K-AKt signaling pathway and pathways in cancer. Based on KME (> 0.8) and gene significance (> 0.2) screening (Fig. 5E), the hub genes of the red module were *KRT18, CTHRC1, POSTN, CDH11, FHL2, SPARC, FN1, MAGED1,* and *FSTL1*, while those of the green module included *WNT11, GLI1, PCDH9, GPC3,* and *L1CAM*. The above-mentioned 14 hub genes were constructed on the PPI network in the STRING database. After removing isolated nodes and node pairs, including seven nodes and eight edges, the Cytoscape plug-in CytoHubba algorithm (MCC) was used to construct a network to identify key genes. Seven hub genes (*CDH11, SPARC, FSTL1, FN1, PCDH9, GPC3,* and *WNT11*) were identified, of which the first four (*CDH11, SPARC, FSTL1,* and *FN1*) belonged to the red module, and the last three (*PCDH9, GPC3,* and *WNT11*) were key genes of the green module. The above-shown results suggest that these EMT-related genes may play a key role in the formation and progression of aneurysms.

### Verification of the hub genes

**Figure 6:**
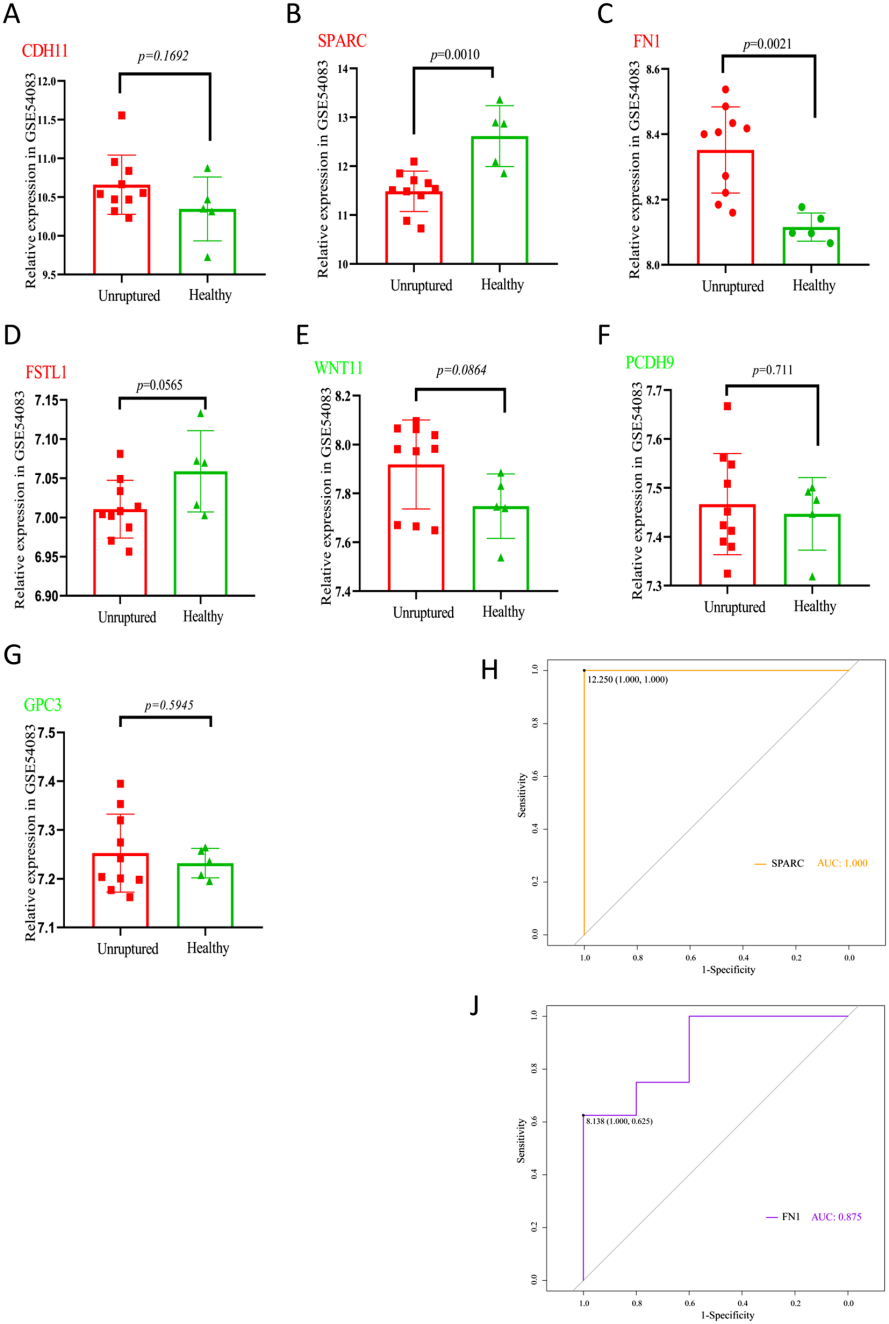
Expression verification of EMT-related key genes. (**A**–**G**) The above-mentioned seven EMT-related genes (*CDH11, SPARC, FN1, FSTL1, WNT11, PCDH9,* and *GPC3*) expressed with relative expression trends in the GSE54083 dataset (unruptured, healthy) were visualized via Graphpad Prism 8.0.0. (**H**–**J**) Receiver operating characteristic (ROC) curves of (*SPARC, FN1*), and the area under the ROC curve (AUC) to evaluate the diagnostic value were visualized by R software 4.0.3.

The above-mentioned seven EMT-related genes were verified in the GSE54083 dataset (external validation dataset) (Fig. 6A–G). It was found that the expression levels of *CDH11, SPARC, FN1,* and *FSTL1* genes in unruptured aneurysms were higher than those in healthy samples, while the expression levels of *WNT11, PCDH9,* and *GPC3* in unruptured aneurysms were lower than those in healthy samples (Fig. 6A–G). In order to further determine the predictive power of the hub gene, we drew receiver operating characteristic (ROC) curves and calculated the area under the ROC curve (AUC) in the GSE54083 dataset (Fig. 6H, I). Results showed that the two genes *SPARC* and *FN1* had AUC of 1.000, 0.875. These two genes may show a good predictive performance in UIAs.

## Discussion

IA is a potentially lethal cerebrovascular disease, which can cause SAH upon rupturing and result in irreparable damage or death. UIAs are usually formed by the destruction of the vascular wall elastic membrane, endothelial damage, and extracellular matrix disorder, and are accompanied by macrophage infiltration and migration^16^. EMT is an important biological process. Specifically, endothelial cells can acquire a mesenchymal phenotype through EMT. The expression of certain targets, including p120-catenin (p120ctn) and endothelial–cadherin^17^, endothelial nitric oxide synthase and superoxide expression^18^, are reprogrammed in EMT. Our study first identified the differential EMT-genes of healthy, ruptured aneurysms and unruptured aneurysms, and demonstrated gene expression patterns. IA is always asymptomatic before rupture, identifying the gene expression pattern of UIAs may reduce the incidence of death in IA patients, and we further explored the gene expression pattern of UIA. The genes related to EMT were determined to be used to perform WGCNA. According to the results, a total of 9 gene modules had been identified, interestingly, we found that the red and green modules were related to UIAs, and the results of PPI and ROC suggested that there were such results that SPARC and FN1 have better specificity and sensitivity. This also means that, in the process of clinical practice, it may be used as a screening method to identify patients with asymptomatic aneurysm and reduce aneurysm rupture.

This study analyzed the cerebrovascular samples from two datasets (GSE13353 and GSE75436); after a pairwise comparison, DEGs were found in the unruptured, ruptured, and healthy groups. Common DEGs (*ADIPOQ, WNT11,* and *CCL21*) were identified, and enrichment analysis of DEGs in different paired groups was performed. GO enrichment analysis of the ruptured and healthy groups revealed that the terms “lipopolysaccharide^19^-induced inflammation” and “activated vascular endothelial cell” were enriched. These play a key role in atherosclerosis or aneurysm formation, including response to molecules of bacteria and KEGG analysis results. This suggests the presence of a chemokine signaling pathway, which may be involved in inflammatory response^20^. The GO and KEGG analysis results in the ruptured and unruptured groups suggested that angiogenesis and epithelial cell proliferation play important roles, possibly via the PI3K-AKT signaling pathway. Various previous studies have also confirmed that angiogenesis^21,22^ of the aneurysm wall requires vascular endothelial cells^23,24^ to coordinate migration, proliferation, and connection formation^25^. Differential gene enrichment analysis between the unruptured and healthy groups showed enrichment for ossification and genitourinary disorders. Arterial calcification is similar to the process of bone organization, relying on the balance of osteoblasts and osteoclasts as well as the arterial regulation of calcium ions by the kidneys. In addition, changes in mechanical stress^26^ after aneurysm formation can regulate the expression of calcification-related genes in vascular endothelial cells and mesenchymal cells^27^; furthermore, endothelial cells can stimulate fibroblasts and smooth muscle cells through cytokine–cytokine receptor interactions. *ADIPOQ* (adiponectin, C1Q, and collagen domain-containing) has been identified as an important gene related to abdominal aortic aneurysm (AAA)^28^. Moreover, adiponectin gene polymorphisms are related to the prognosis of aneurysmal subarachnoid hemorrhage^29^. *WNT11* (Wnt family member 11) has been reported in endothelial cells that cope with vascular shearing force. The absence of WNT11 makes it easier for endothelial cells to withstand shearing force and keep blood vessels open^30^. A study by Guedj et al^31^ confirmed that the inflammatory factor *CCL21* (C–C motif chemokine ligand 21) in human abdominal aortic aneurysms can trigger smooth muscle cells to produce chemokines, thereby aiding in the recruitment of immune cells and development of atherosclerosis. Krupa et al.^32^ revealed that adventitial CD83+ dendritic cells, as key antigen-presenting cells, produce the chemokines CCL19 and CCL21 after maturation, which may lead to aortic aneurysms. There is no doubt that EMT activity is associated with various pathological processes, including the migration of vascular endothelial cells and the activity of inflammatory cells. These findings reveal the mechanisms potentially involved in the formation and rupture of aneurysms, as well as important and key EMT-related genes.

In this study, key genes were calculated using the limma and WGCNA algorithms. limma is an R/Bioconductor package^13^, which is a tool for analyzing gene expression data in related samples and obtaining differential expression schemes. WCGNA can analyze gene expression patterns of multiple samples to cluster genes with similar expression patterns and analyze the associations between modules and specific traits or phenotypes. Therefore, WGCNA is widely used in research on diseases and other traits and gene association analysis.

Through WGCNA construction, we found that the unruptured group (red and green modules) and the healthy group (red and green modules) showed opposite correlation patterns. Therefore, we speculate that the expression of genes in these two modules plays a key role in the formation and occurrence of aneurysms. The genes in the red module (*KRT18, CTHRC1, POSTN, CDH11, FHL2, SPARC, FN1, MAGED1,* and *FSTL1*) and the green module (*WNT11, GLI1, PCDH9, GPC3,* and *L1CAM*) can be regarded as hub genes of UIAs. They played an important role in certain co-expression networks. However, their roles in the aneurysm formation mechanism remain unclear. We selected upregulated genes (*CDH11, SPARC, FN1,* and *FSTL1*) and downregulated genes (*WNT11, PCDH9,* and *GPC3*) from the perspective of PPI. CDH11 (cadherin 11), a mediator of intimal hyperplasia, regulates vascular smooth muscle cells and plays an important role in vascular remodeling^33^. It has been reported that secreted protein acidic and cysteine-rich (*SPARC*) is highly expressed in intracranial aneurysms and is associated with the expression of EMT-related molecules (namely *MMP2* and *MMP9*). It may mediate changes in the intracranial aneurysm extracellular matrix^34^. This finding is consistent with our results. *FN1* (fibronectin 1) is an important part of the artery extramural matrix, which is crucial for maintaining the integrity of the arterial wall^35^. Gorelik et al. ^36^ found that the expression of *FSTL1* (follistatin-like 1) is related to the incidence of Kawasaki disease and can predict the formation of coronary aneurysms. Pénisson-Besnier^37^ reported another *GPC3* (glypican 3) gene exon 8 mutation, which may be the cause of carotid artery dissection. In our other dataset (GSE54083), we were surprised to find that the expression of *CDH11, SPARC, FN1,* and *FSTL1* in unruptured aneurysms was significantly increased, while *WNT11*, *PCDH9*, and *GPC3* expression levels were relatively low. These genes (*SPARC*, and *FN1*)showed high sensitivity and specificity according to the ROC results. Indeed, most of the mechanisms of these EMT-related genes in aneurysms remain unclear. our future research is necessary to explore the potential biological functions of the above-mentioned EMT-related genes.

Our study had some limitations. The clinical samples we included were limited, and a larger aneurysm cohort is needed to confirm our results. In addition, our results are based on bioinformatic analysis, and limitations of surgical methods used in our hospital made it difficult to collect clinical tissue samples for verification. Therefore, we could not further explore the changes in the expression of these genes in patients over the course of their disease. However, our results are still instructive for the diagnosis and prediction of aneurysms. In the future, our research will focus on this aspect, hoping to gain deeper experience in the management of aneurysms.

## Supplementary Information

Supplementary Information 1.

Supplementary Information 2.

## Data Availability

The datasets analyzed in this study are available in the Gene Expression Omnibus (GEO) public repository. KEGG materials were obtained the formal permission of Kanehisa laboratories. The data used to support the results of this study can be obtained from the corresponding author.
