# Supplementary material for: Epithelial–mesenchymal transition related genes in unruptured aneurysms identified through weighted gene coexpression network analysis
Source: Sci Rep. 2022 Jan 7;12:225. doi: 10.1038/s41598-021-04390-6 (PMC8741966; doi:10.1038/s41598-021-04390-6)
Supplement: Supplementary file 1 — Supplementary Information 1. [file 41598_2021_4390_MOESM1_ESM.pdf]

Epithelial mesenchymal transition related genes in unruptured aneurysms identified through weighted gene coexpression network analysis

Yong'an Jiang [1], JingXing Leng[1,*],QianXia Lin [2], Fang Zhou [3,*]

Affiliations:

[1]Department of Neurosurgery, Jiangxi provincial People's hospital affiliated to Nanchang University, Nanchang, Jiangxi,330006, China,

[2]Jiangxi University of Traditional Chinese Medicine, Nanchang, Jiangxi,330006, China,

[3]Vascular Breast Surgery, Jiangxi provincial People's hospital affiliated to Nanchang University, Nanchang, Jiangxi,330006, China,

[*]Corresponding Author,

Yong'an Jiang [1] and JingXing Leng[1,*] contribute equally to this article.

Figure S1: research procedure.

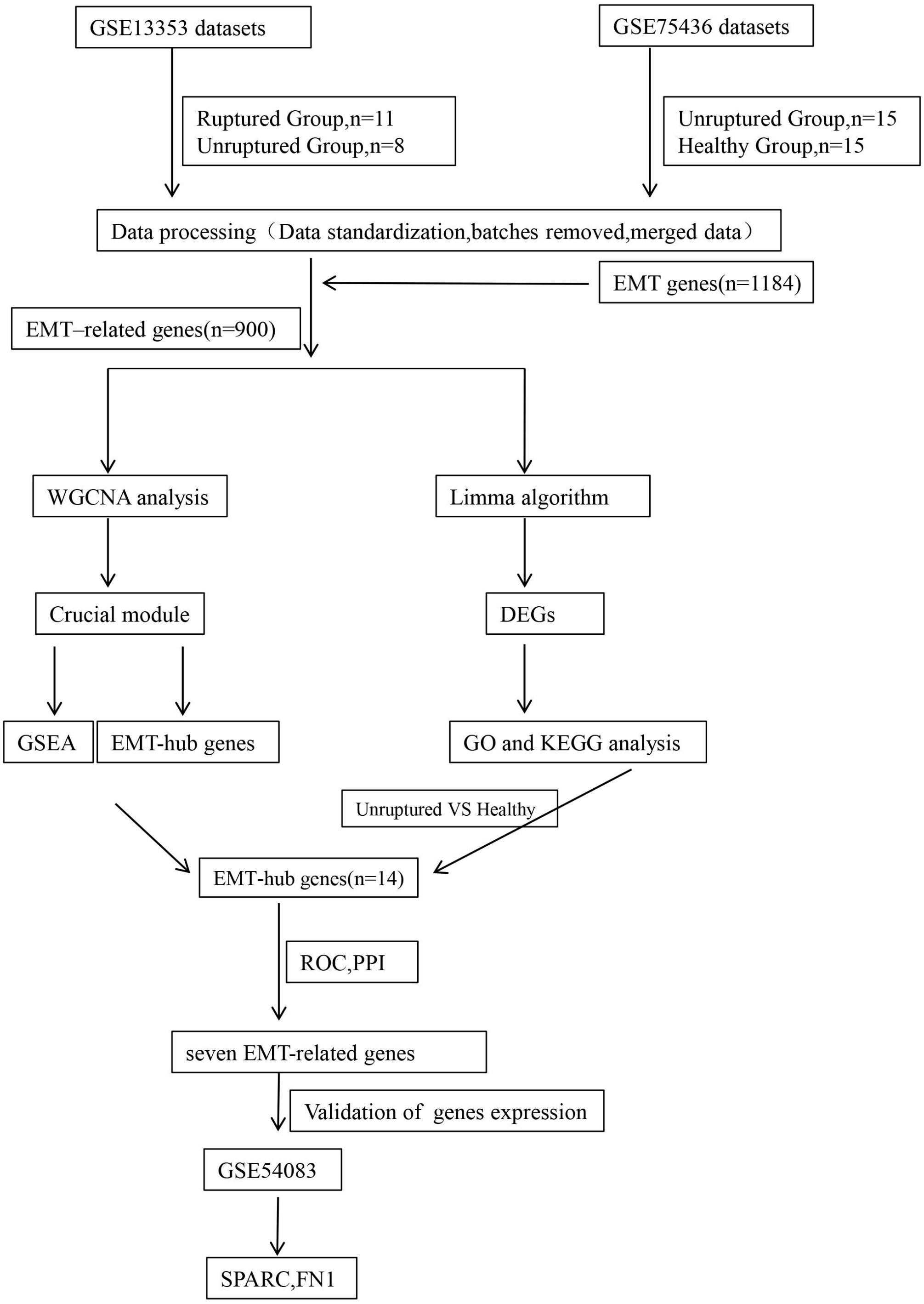

Figure S2:

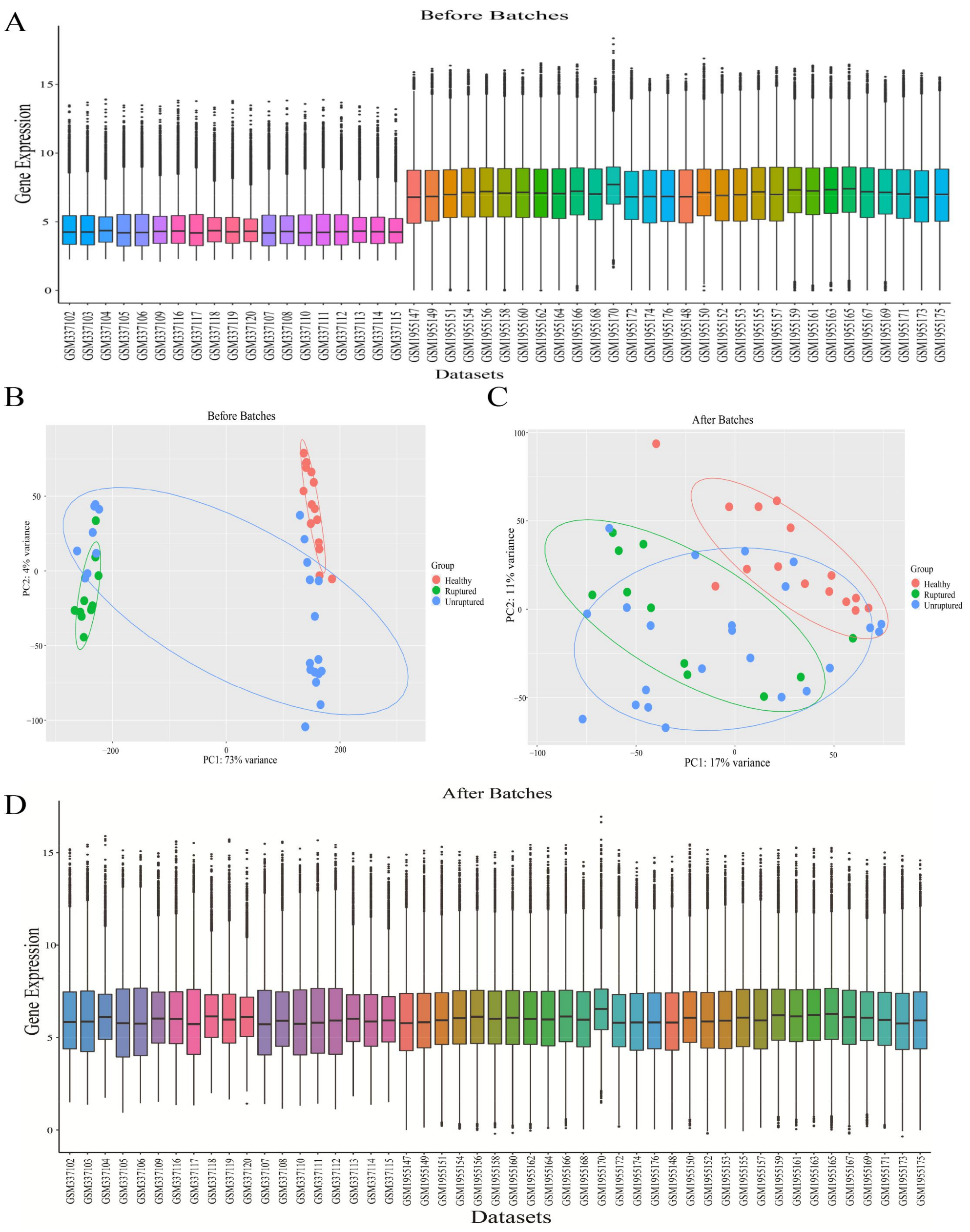

Figure S2: Merged datasets and removed batch effects.**A.** Datasets (GSE13353, GSE75436) standardized process, before removing batches. **B.** Principal Component Analysis(PCA) before removing batches. **C.** PCA analysis after removing batches. **D.**after removing batches.above all figures were visualized by R software 4.0.3.

Figure S3:

A

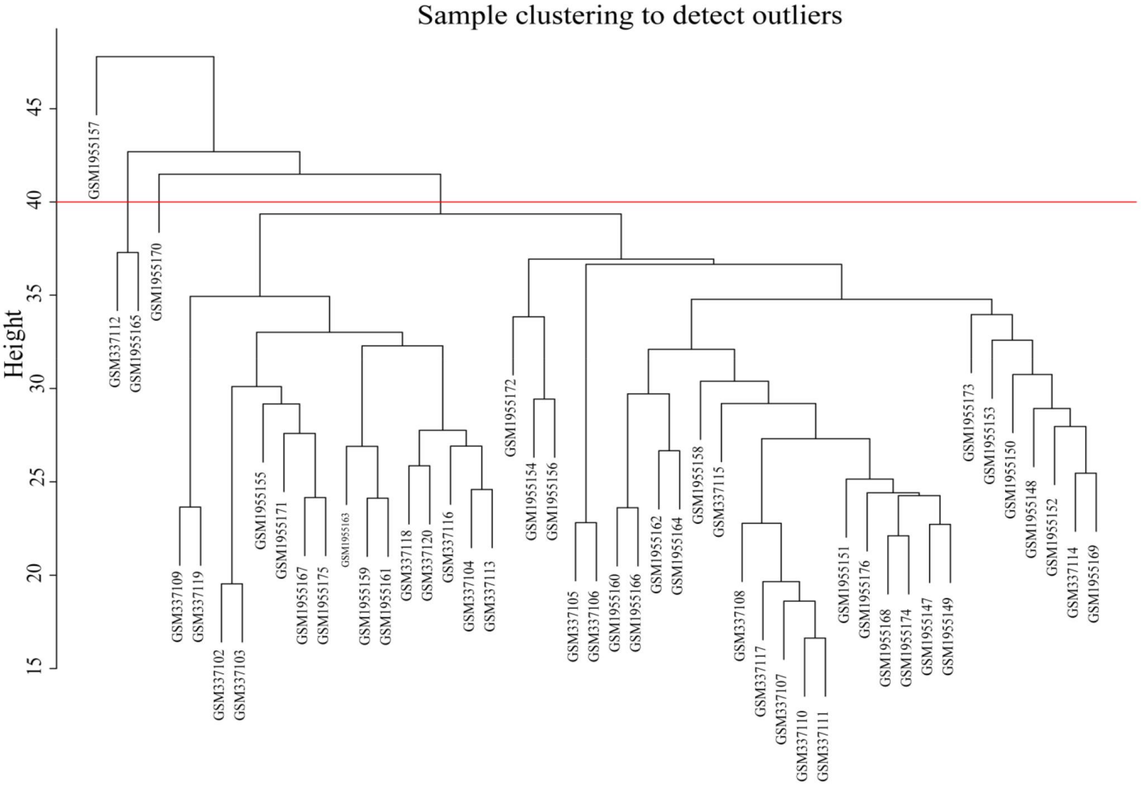

Figure S3: A. Sample clustering screened out outliers(red line)=40.above all figures were visualized by R software 4.0.3.

Figure S4:

A

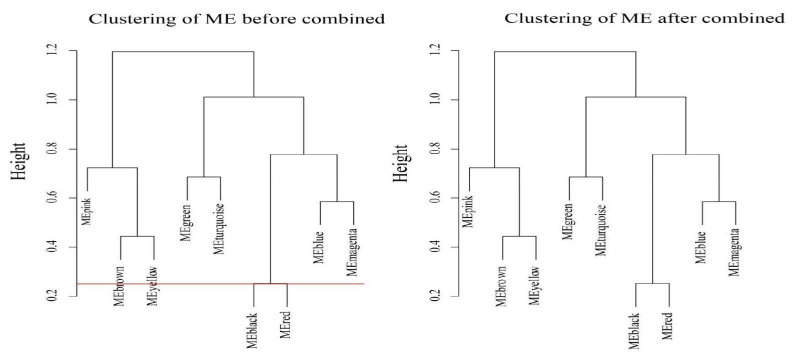

B

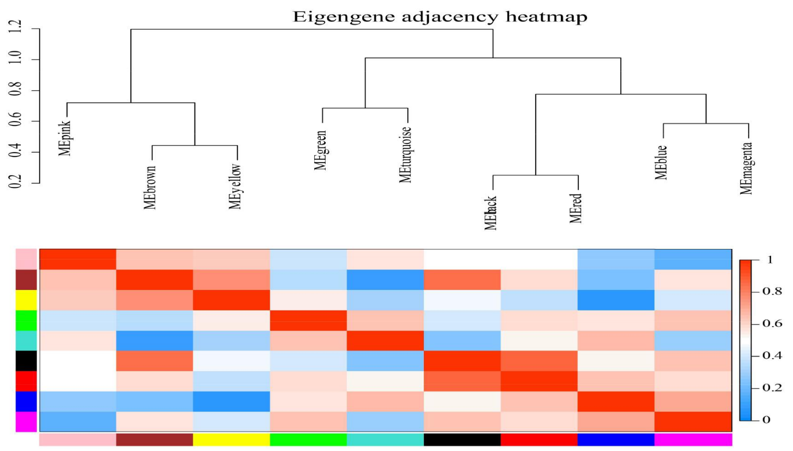

C

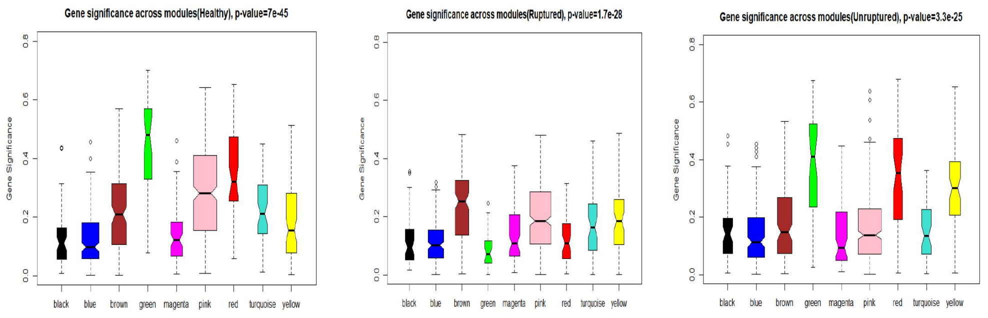

Figure S4: A. (Before being combined) The dendrogram of the characteristic genes of the consensus module obtained via WGCNA on the consensus correlation. The red line is the merge threshold (0.25). Eigengenes below the threshold indicate the modules whose expression profiles should be merged due to similarity. B. Clustering of MEs after being combined. **B.** The relationship between the module and neighboring modules. Red represents high correlation, and blue represents negative correlation. C. The important modules in the three groups (unruptured, ruptured, healthy) are represented by box diagrams. above all figures were visualized by R software 4.0.3.

Table S1： information on samples and authors of datasets (GSE13353, GSE75436, GSE54083)

datasets	samples	Platforms	Contributor(s)
GSE13353	aneurysm_ruptured(n=11); aneurysm_unruptured(n=8)	GPL570	Yu L et.al[1]
GSE75436	aneurysm_unruptured(n=15); superficial temporal artery(n=15)	GPL570	Kurki MI et.al[2]
GSE54083	Ruptured intracranial aneurysm(n=8); Unruptured intracranial aneurysm(n=5); Superficial temporal artery(n=10)	GPL4133	Inoue I et.al[3]

1 Kurki, M. I. *et al.* Upregulated signaling pathways in ruptured human saccular intracranial aneurysm wall: an emerging regulative role of Toll-like receptor signaling and nuclear factor-κB, hypoxia-inducible factor-1A, and ETS transcription factors. *Neurosurgery* **68**, 1667-1675; discussion 1675-1666, doi:10.1227/NEU.0b013e318210f001 (2011).

2 Wang, W. *et al.* Aberrant expression of lncRNAs and mRNAs in patients with intracranial aneurysm. *Oncotarget* **8**, 2477-2484, doi:10.18632/oncotarget.13908 (2017).

3 Nakaoka, H. *et al.* Gene expression profiling reveals distinct molecular signatures associated with the rupture of intracranial aneurysm. *Stroke* **45**, 2239-2245, doi:10.1161/strokeaha.114.005851 (2014).

Table S2: DEGs of (Unruptured VS Healthy, Ruptured VS Unruptured and Ruptured VS Healthy).

DEG_Unruptured_Healthy	symbol	logFC	Pvalue	direction
	ADIPOQ	-3.414312601	1.04E-10	Down
	WNT11	-2.490269899	1.18E-10	Down
	GPC3	-1.701181	8.73E-10	Down
	CCL21	-2.840780977	4.90E-08	Down
	CEMIP	2.879414244	2.51E-07	Up
	CDKN2A	1.389749422	2.79E-07	Up
	HAS2	-1.46907007	2.85E-07	Down
	AGTR1	-1.94339542	4.99E-07	Down
	TNC	-2.503298935	1.81E-06	Down
	CDH2	1.775682567	1.95E-06	Up
	CDH11	1.665029441	2.09E-06	Up
	GLI1	-1.810649614	2.21E-06	Down
	SALL1	2.31139036	4.21E-06	Up
	CXCL14	-2.527971286	5.02E-06	Down
	CTHRC1	1.996241729	7.82E-06	Up
	VCAN	1.696766658	8.49E-06	Up
	SDC1	1.379015828	9.15E-06	Up
	L1CAM	-1.303169011	9.98E-06	Down
	LEFTY1	-1.332973791	2.03E-05	Down
	SEMA3E	-1.654174731	3.61E-05	Down
	PDGFD	-1.097233622	5.48E-05	Down
	SOX17	-1.290679344	6.11E-05	Down
	KRT18	2.042998207	6.77E-05	Up
	CLDN7	1.491705712	9.75E-05	Up
	TNF	1.455946393	0.000131612	Up
	PTN	1.568125807	0.000137237	Up
	ISG15	1.445825957	0.000171209	Up
	NF1	-1.007068535	0.000176212	Down
	IL1B	1.577071573	0.000184751	Up
	CRMP1	1.542459453	0.000202571	Up
	LAMA1	1.238790341	0.000241835	Up
	ADAM12	1.667261493	0.000245338	Up
	AOC4P	-1.239395524	0.000265903	Down
	CD36	-1.72395086	0.000270276	Down

WNT5A	1.488664713	0.000278884	Up
VSNL1	1.440534975	0.000279326	Up
POSTN	1.5405255	0.000299393	Up
PDPN	1.024021369	0.00035859	Up
TLR4	1.043221964	0.00048203	Up
IL18	1.38552503	0.000722122	Up
LEP	-1.891124869	0.000735556	Down
SERPINE1	1.424864926	0.000735717	Up
RBP2	-1.021140801	0.000781664	Down
CCR5	1.497903371	0.000812172	Up
HAVCR2	1.120219955	0.000843781	Up
KLF5	-1.305651546	0.001228823	Down
HOXB7	1.047121093	0.001375249	Up
CCL19	-1.467900815	0.001536093	Down
CXCL16	1.433998972	0.001723536	Up
SPRY2	-1.032049561	0.001817461	Down
ROR2	1.292879485	0.001855635	Up
TNFAIP8L2	1.107666433	0.001877938	Up
SPP1	2.078169546	0.001908017	Up
PLAC8	1.419875765	0.002200013	Up
ACTG2	-1.257230125	0.002256496	Down
IRF8	1.07788524	0.002631756	Up
ALDH1A1	-1.159208631	0.002997022	Down
MMP13	1.08828474	0.003911253	Up
P2RY2	-1.182187035	0.005198312	Down
FOXQ1	1.260314723	0.005245344	Up
VSIG4	1.548411145	0.005424095	Up
KIT	1.112751337	0.005456265	Up
CD14	1.561482419	0.005870081	Up
FLNA	-1.121623804	0.005978445	Down
MYL2	-1.862430795	0.006305062	Down
MYC	-1.141033958	0.006599565	Down
CRYAB	-1.064617998	0.006680457	Down
SERPINI1	-1.037613267	0.007073019	Down
CCL20	1.989521867	0.00758511	Up
HMOX1	1.77805992	0.008235887	Up
GREM1	1.585070789	0.010283712	Up
CXCR4	1.15675893	0.014828955	Up
DKK1	1.041438727	0.015225929	Up
CCL18	2.072846104	0.017414638	Up
C5AR1	1.080511394	0.029110488	Up
IL6	-1.544384641	0.030116074	Down
CXCL8	1.3231133	0.049808489	Up

DEG_Ruptured_Unruptured	symbol	logFC	Pvalue	direction
	CD36	1.945101497	0.000206825	Up
	NUAK1	-0.94943931	0.000439436	Down
	WNT11	1.268145203	0.00048186	Up
	HAS2	0.98638323	0.000722536	Up
	PDGFD	0.96185361	0.000986021	Up
	MYC	1.536432188	0.001128997	Up
	ADIPOQ	1.577940183	0.00131497	Up
	SOCS3	1.083689396	0.001438089	Up
	CDH11	-1.081108058	0.002724222	Down
	DLX2	-0.990401426	0.002769707	Down
	SPHK1	1.013904587	0.003644024	Up
	TNC	1.552301435	0.003809356	Up
	FZD7	-1.286224076	0.004031134	Down
	IL6	2.291991335	0.00421511	Up
	CCL21	1.444246502	0.004762857	Up
	KDM6B	0.945487596	0.007679641	Up
	VCAN	-1.03411864	0.008538159	Down
	SALL1	-1.323086693	0.010023533	Down
	LEP	1.544704789	0.010459407	Up
	PTX3	1.718303865	0.011131976	Up
	ANGPTL4	1.32315373	0.011801521	Up
	THBD	0.925711539	0.012420975	Up
	SERPINF1	1.247922064	0.013413321	Up
	ITGA5	1.100361505	0.014447205	Up
	CXCL14	1.349961692	0.016848401	Up
	NAMPT	1.126017316	0.017694818	Up
	KLF4	0.883921855	0.018140427	Up
	ADM	1.108970845	0.019077506	Up
	SEMA3E	0.944285076	0.023061995	Up
	NFIL3	0.90208518	0.024528402	Up
	PLAUR	1.069383831	0.026728759	Up
	TIMP1	0.978877374	0.029377665	Up
	KLF5	0.911396664	0.035105954	Up
	ID1	-0.917335346	0.036484394	Down
	CXCR2	1.224816936	0.03861071	Up
	P2RY2	0.946075169	0.039195903	Up
	CXCL8	1.53610638	0.039620311	Up
	AQP9	1.584584323	0.040862962	Up
	S100A8	1.661108205	0.041288341	Up
	CCL2	1.253207254	0.043680305	Up

	MMP9	1.095566347	0.045590557	Up
	NTRK3	-0.867817826	0.04754577	Down
	S100P	1.25551415	0.049129356	Up
DEG_Ruptured_Healthy	symbol	logFC	Pvalue	direction
	SDC1	1.543680353	2.69E-05	Up
	HK2	1.095821385	0.000137303	Up
	TIMP1	1.911106745	0.000182303	Up
	HAVCR2	1.515700847	0.000197324	Up
	CCR5	1.946081957	0.000313916	Up
	CXCL16	1.982905745	0.000359151	Up
	HMOX1	2.956111974	0.000364578	Up
	LYN	1.417782685	0.000398022	Up
	CXCR4	2.066229649	0.000434	Up
	CD14	2.442080676	0.000441761	Up
	MYD88	1.006080403	0.000457164	Up
	CSK	1.055906157	0.000485265	Up
	CEMIP	2.14863231	0.000504104	Up
	SERPINI1	-1.616633098	0.000609957	Down
	ADIPOQ	-1.836372418	0.000609997	Down
	CXCL8	2.85921968	0.000662891	Up
	AGTR1	-1.460673116	0.000674226	Down
	SERPINE1	1.717181586	0.000676964	Up
	C5AR1	2.037771994	0.000870558	Up
	CTSL	1.409488183	0.000904003	Up
	ADAM12	1.777609744	0.00092708	Up
	AFAP1L2	-1.062601665	0.000967754	Down
	CCL20	2.998201013	0.000970718	Up
	SPP1	2.644095624	0.00102916	Up
	FBP1	1.381060285	0.001046778	Up
	VSIG4	2.21364601	0.00106618	Up
	TLR4	1.143435059	0.001245014	Up
	ABCC3	1.32571505	0.001288235	Up
	ST14	1.504750417	0.001362979	Up
	GRN	1.391153261	0.001387686	Up

IRF8	1.366776367	0.001514877	Up
TNFAIP8L2	1.354146948	0.001518536	Up
WNT5A	1.524045712	0.001559673	Up
WNT11	-1.222124696	0.00166496	Down
PLAUR	1.682940058	0.001698678	Up
DAB2	1.132975405	0.00200041	Up
CTSZ	1.042885601	0.002003093	Up
NLRP3	1.23623685	0.002017872	Up
MCAM	-1.029087359	0.002319889	Down
AR	-1.194539637	0.002337149	Down
DKK1	1.527847249	0.00333489	Up
MMP19	1.131602523	0.003651876	Up
PTPN6	1.124565098	0.004313693	Up
CLDN7	1.258093056	0.004351941	Up
CRYAB	-1.327079248	0.004829505	Down
PHLDA1	1.005577178	0.005132826	Up
POSTN	1.370587869	0.00565866	Up
IL18	1.320357003	0.005980773	Up
CCL5	1.556570486	0.006182523	Up
PSTPIP1	1.059045966	0.006969773	Up
NTRK3	-1.297223429	0.007165658	Down
CAV1	-1.156720569	0.007804753	Down
TACC3	1.331605	0.007827392	Up
CCL18	2.786820749	0.007962084	Up
PRKAA2	-1.086418221	0.0085121	Down
PLAC8	1.403258371	0.010264892	Up
ACTA2	-1.404907204	0.010472398	Down
CCL21	-1.396534475	0.011071656	Down
CDH2	1.030467045	0.011816487	Up
GREM1	1.849451682	0.012170229	Up
UCP2	1.23827826	0.01387672	Up
HPSE	1.115539889	0.015386041	Up
CYP1B1	1.126984303	0.017142347	Up
ROR2	1.135786973	0.019456908	Up
PCDH9	-1.067607421	0.021913941	Down
EMP3	1.005252626	0.022357887	Up
AQP9	1.923867068	0.022558476	Up
FLNA	-1.092100615	0.023452776	Down
CTHRC1	1.111123612	0.024430974	Up
ANGPT	1.260350988	0.025704714	Up

L4			
IL1B	1.070194608	0.026166817	Up
LIF	1.130484289	0.02795256	Up
FOXM1	1.036298268	0.030257012	Up
MMP1	1.986151642	0.031094204	Up
PTN	1.004203068	0.0315546	Up
MMP9	1.245671474	0.036175181	Up
